# 18S/28S rDNA metabarcoding identifies *Cryptosporidium parvum* and *Blastocystis* ST1 as the predominant intestinal protozoa in hospital patients from Changchun, Northeast China

**DOI:** 10.1186/s13071-025-07043-z

**Published:** 2025-09-24

**Authors:** Cunmin Wang, Jigang Yin, Zhanpeng Shi, Yijia Xu, Junhong Chen, Yueyang Yan, Guan Zhu, Jixue Zhao

**Affiliations:** 1 State Key Laboratory for Diagnosis and Treatment of Severe Zoonotic Infectious Diseases, Key Laboratory for Zoonosis Research of the Ministry of Education, Institute of Zoonosis, College of Veterinary Medicine, Jilin University, Changchun, 130062 Jilin China; 2 Department of Pediatric Surgery, The First Hospital of Jilin University, Changchun, 130021 Jilin China

**Keywords:** *Cryptosporidium parvum*, *gp60* IIdA19G1, *Blastocystis* ST1, Metabarcoding, Pooled sampling, Northeast China, Intestinal parasites, RDNA amplicons

## Abstract

**Background:**

Intestinal protozoa and helminths remain an under‑recognized cause of gastrointestinal morbidity in China. Molecular high‑throughput tools offer the chance to survey their diversity comprehensively, yet their application in clinical settings has been limited.

**Methods:**

We pooled leftover fecal samples from 360 hospital patients in Changchun (36 pools; 12 demographic/seasonal groups) and enriched them by sucrose flotation. Three primer pairs targeting 18S V4‑V5, 18S V9 and 28S D3‑D4 rRNA regions were amplified, and paired‑end libraries (100–140 k reads per amplicon) were sequenced on Illumina platforms. Taxa were assigned with QIIME2 against SILVA, and true prevalences were estimated from pooled‑sample data using a binomial model with profile‑likelihood confidential intervals. Selected positives were confirmed by qPCR, nested PCR, *gp60* subtyping and immunofluorescence assay.

**Results:**

From 6.1 million quality‑filtered reads, only 1.65% mapped to parasites; fungal reads dominated (98.35%), underscoring primer bias. Four eukaryotic parasites were detected across 12/36 pools. *Cryptosporidium parvum* was most frequent (7 pools, true prevalence = 2.14%, 95% CI 0.92–4.10), and all *gp60*‑typed isolates belonged to subtype IIdA19G1. *Blastocystis hominis* occurred in five pools (1.48%, 0.53–3.17), predominantly ST1, with single detections of ST3 and ST6. *Entamoeba hartmanni* appeared in one pool (0.28%, 0.02–1.23). Reads assignable only to Opisthorchiidae suggested liver‑fluke carriage in four adult pools (1.17%, 0.36–2.70). No statistically significant associations were found between infection status and age, sex, season or diarrhea. Amplification success differed markedly between primer sets, limiting quantitative comparisons.

**Conclusions:**

Metabarcoding of rDNA amplicons provides a feasible snapshot of human intestinal‑parasite communities in Northeast China, revealing *C.* *parvum* IIdA19G1 as an emerging zoonotic threat and highlighting ongoing food‑borne trematodiasis. However, the overwhelming amplification of fungal templates and inter‑primer bias call for primer redesign and complementary diagnostics before routine clinical adoption.

**Graphical Abstract:**

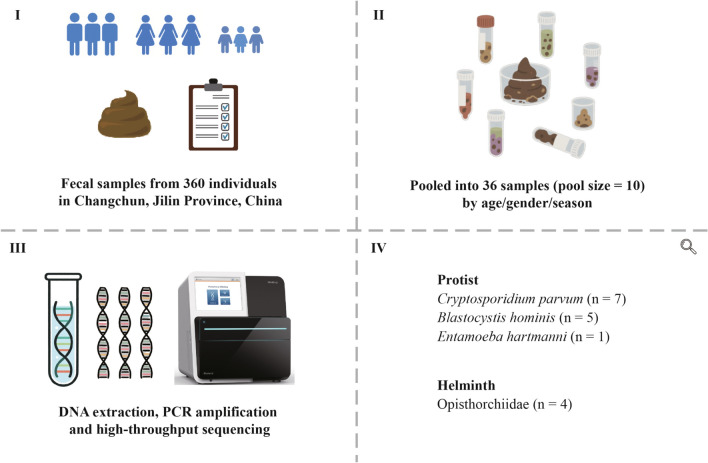

**Supplementary Information:**

The online version contains supplementary material available at 10.1186/s13071-025-07043-z.

## Background

Intestinal parasites, including protists and helminths, remain a significant public health concern worldwide, affecting both humans and animals. In humans, these infections are generally more prevalent and severe in resource-limited settings, while vulnerable populations such as children and immunocompromised individuals are also at risk even in developed regions [1–3]. Opportunistic parasites like *Cryptosporidium* and *Toxoplasma* are particularly common in these groups [2, 4]. Moreover, several intestinal parasites, including *Giardia*, *Cryptosporidium*, *Toxoplasma* and various nematodes and cestodes, are responsible for waterborne and foodborne outbreaks globally [5–8]. In animals, intestinal parasitic diseases are widespread in both livestock and wildlife, leading to considerable economic losses in agriculture. For example, coccidiosis in poultry, cryptosporidiosis in calves and various helminthiases in cattle, pigs and small ruminants remain major concerns [9–12].

Globally, the burden of human parasitic diseases, including intestinal parasites, differs significantly by region. Developing countries face a high burden of widespread soil-transmitted helminths, water-, food- and vector-borne parasites, while developed countries encounter various water-, food- and vector-borne parasites, although typically at lower prevalence rates and often linked to specific risk factors like travel or localized outbreaks [13, 14]. In China, a country with a vast population and geographic diversity, significant progress has been made in the control and elimination of parasitic diseases through government-led initiatives, public health campaigns and multi-sectoral strategies [15]. These efforts have notably reduced the burden of diseases such as schistosomiasis, soil-transmitted helminthiases, clonorchiasis and cysticercosis. However, despite these achievements, intestinal parasitic infections persist, particularly in marginalized regions characterized by poverty, traditional dietary practices or close human-animal interactions [16, 17]. Sustainable parasite control will require integrated approaches involving sanitation, education, food safety and One Health-based surveillance.

Epidemiological surveys remain essential for assessing parasite prevalence and informing public health policy. However, data on the current prevalence and diversity of intestinal parasites in humans in China remain fragmented and sparse. A review of PubMed-indexed Literature from the past decade identified 159 original studies that addressed intestinal parasites in China, in which the majority focused on animal hosts (Table S1). Only around 20% of the studies addressed human infections, and most of these studies used PCR-based detection targeting one or a few predefined species, limiting the assessment of parasite community composition. To date, only a single study has applied metabarcoding to intestinal parasites in China, detecting multiple protozoa and helminths in black-necked cranes [18].

High-throughput sequencing methods, such as metagenomics and metabarcoding, offer powerful tools to investigate complex parasite communities without prior assumptions about species presence [19, 20]. These methods can detect low-abundance organisms and assess overall community structure and diversity. Although widely applied in microbiome and virome research, they remain underutilized for studying eukaryotic microbes, including parasites, largely due to the low abundance, large and diverse genomes, and host DNA contamination common in clinical and environmental samples. Nonetheless, rRNA gene-targeted metabarcoding using universal primers for 18S and 28S rDNA has shown promise in detecting intestinal parasites when carefully optimized [21, 22].

In this study, we employed a metabarcoding approach to survey intestinal parasites in human patients visiting a teaching hospital in Changchun, a major city in northeast China. Using three previously validated primer sets targeting 18S (*n* = 2) and 28S (*n* = 1) rRNA genes, we amplified DNA from 36 pooled fecal samples (*n* = 360 individuals) and analyzed them using next-generation sequencing. Our results identified three intestinal protists, *Cryptosporidium parvum*, *Entamoeba hartmanni* and *Blastocystis hominis*, as well as a liver fluke. This study offers a snapshot of the human intestinal parasite landscape in northeastern China and demonstrates the feasibility of metabarcoding for parasite surveillance in clinical samples.

## Methods

### Patient cohorts and fecal sample collection

This study analyzed leftover fecal samples (*n* = 360) collected from consecutive patients at Jilin University First Hospital between September 2023 and April 2024, originally submitted for routine clinical diagnosis and available in sufficient quantity for further analysis (Table S2). Samples were obtained from two major patient groups: (i) children and adolescents with diarrhea (0 to 17 years old, *n* = 120), predominantly outpatients (*n* = 118) from the Pediatric Gastroenterology Department, and two inpatients at the Gastroenterology Department; (ii) adults without diarrhea (≥ 18 years old, *n* = 240), mostly inpatients (*n* = 234) from various departments, primarily hepatobiliary and pancreatic medicine (*n* = 109), interventional therapy (*n* = 40), gastroenterology (*n* = 33), oncology (*n* = 16) and cadre ward (*n* = 13). Adult patients were further stratified into middle-aged (18 to 60 years old, *n* = 120) and elderly (≥ 61 years old, *n* = 120) subgroups. The difference in sample size between pediatric patients with diarrhea and adults without diarrhea reflects the higher frequency of diarrhea in children and a greater tendency for caregivers, such as parents, childcare workers or teachers, to seek hospital care for pediatric diarrhea, whereas adults are less likely to seek medical attention for short-term gastrointestinal symptoms.

**Fig. 1:**
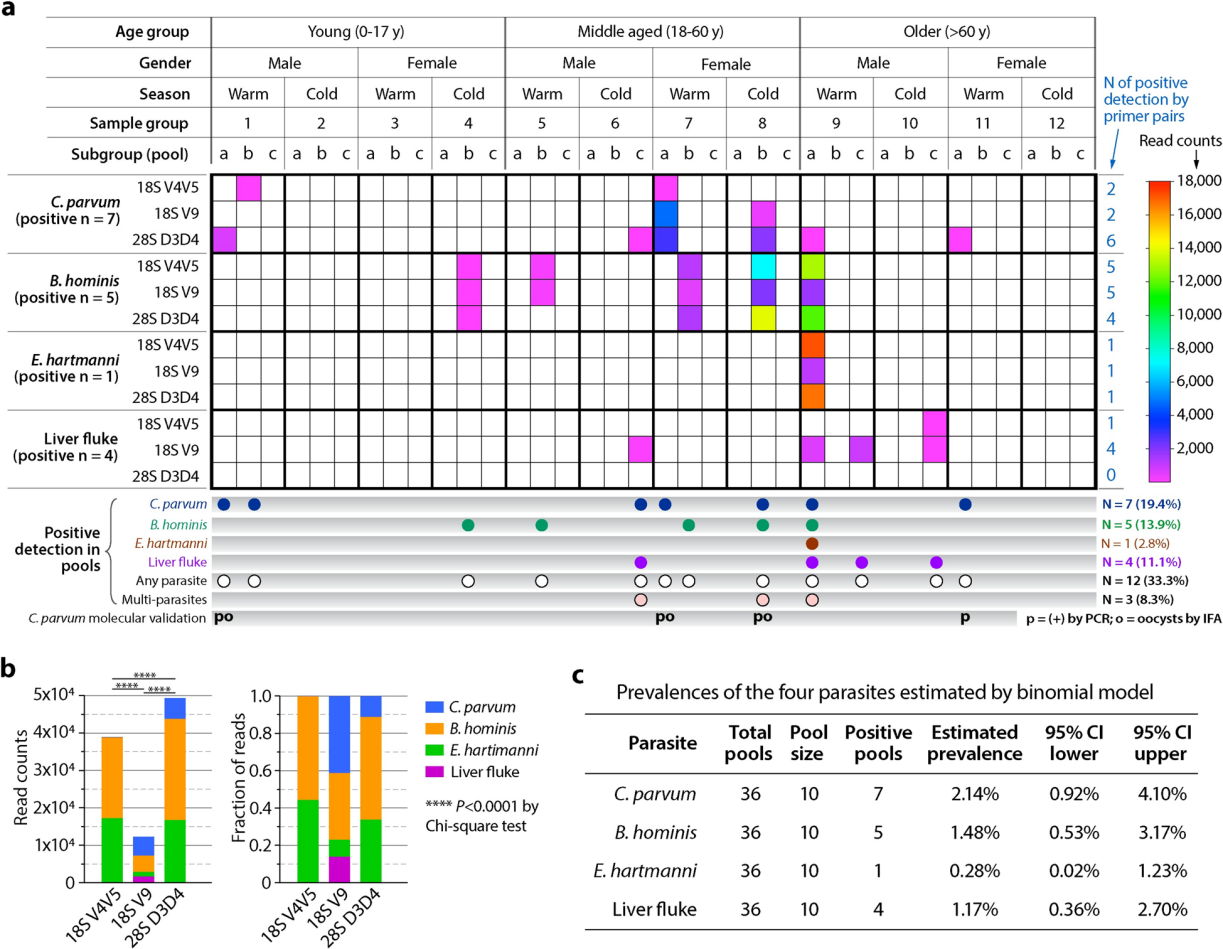
Summary of metabarcoding results for the detection of intestinal parasites in pooled samples (pool size = 10) from human patients. **a** Heatmap showing the detection of the four specified parasites in individual pools by three pairs of primers targeting various 18S and 28S rDNA regions, including 18S rRNA V4V5 region by primers 616*F/1132R, 18S rRNA V9 region by primers 1391F/EukBr and 28S rRNA D3D4 region by primers DM568F/RM2R. Bottom panel summarizes the overall positive detections of individual parasites by metabarcoding as well as by regular PCR or immunofluorescence assay (IFA) of oocysts. **b** Bar charts showing the total read counts or ratios of the total read counts between the four parasites by targeted 18S/28S regions. **c** Positive detection rates for the four parasites in the 36 pools and estimated true positivity rates

To reduce the costs while maintaining statistical power, individual fecal samples were combined into pooled samples (*n* = 36; 10 individuals per pool). These pooled samples are hereafter referred to as ‘pools.’ The pools were assigned to 12 groups stratified by age (young [Y], middle [M], older [O]), gender (male [M], female [F]) and season (warm [W], cold [C], based on local climate). Each group contained three pools as biological replicates (Fig. 1 and Table S2).

### Sample preparation and DNA extraction

For each subgroup, 200 mg/patient of wet solid feces from 10 individual samples was thoroughly mixed in a total of 20 ml phosphate-buffered saline (PBS, pH7.2). Pooled samples were homogenized, filtered with a 0.1-mm mesh to remove large debris, resuspended in 20 ml PBS, layered onto 20 ml sucrose solution (~ 2.4 M in ddH_2_O; specific gravity 1.30–1.35) and centrifuged at 1000 g for 10 min. Materials at the PBS/sucrose interface (~ 5 ml) were carefully transferred to 50-ml tubes, mixed with 20 ml PBS and washed twice in PBS by centrifugation (2000 g for 20 min) [23]. After final centrifugation, the pellets were resuspended in 1 ml lysis buffer and subjected to 10 freeze-and-thaw cycles in Liquid nitrogen and 37 °C water bath to rupture oocyst walls. Samples were mixed with 2-mm stainless steel beads and further ruptured in TissueLyser II (Qiagen, Hilden, Germany) at 30 Hz for 2 min. The suspensions were subjected to SevenEasy DNA Gel Extraction Kit (Seven/Abcels, Beijing, China) following the manufacturer's protocol. Reagent negative controls (extraction blanks) used nuclease-free water processed alongside samples in each batch of extraction to monitor potential contamination during DNA extraction. The concentrations and purity of extracted DNA were evaluated by measuring the 260/280-nm absorbance ratio in a NanoDrop NC2000 spectrophotometer (Thermo Fisher Scientific, MA, USA). Purified DNA was diluted with nuclease-free water to 40 ng/μl and stored at − 40 °C until use.

### Amplification and next-generation sequencing (NGS) of 18S and 28S rDNA amplicons

PCR amplification, library construction and next-generation sequencing (NGS) of 18S and 28S rRNA amplicons were performed by Shanghai Personal Biotechnology (Shanghai, China). Based on previous validation for detecting intestinal parasites [21], the following three pairs of pan-eukaryotic primers targeting 18S/28S rRNA gene (rDNA) were used to produce amplicons for metabarcoding: 616*F (5'-TTAAARVGYTCGTAGTYG-3') and 1132R (5'-CCGTCAATTHCTTYAART-3') targeting the 18S rRNA V4–V5 region (product = 509 bp; in reference to AF040725 for *C. parvum* 18S/28S genes), 1391 F (5'-GTACACACCGCCCGTC-3') and EukBr (5'-TGATCCTTCTGCAGGTTCACCTAC-3') targeting the 18S rRNA V9 region (product = 147 bp) and DM568F (5'-TTGAAACACGGACCAAGGAG-3') and RM2R (5'-TTCGATTRGTCTTTCGCCCCT-3') targeting the 28S rRNA D3–D4 region (product = 292 bp). PCR was performed using the Q5 High-Fidelity DNA Polymerase (New England Biolabs, MA, USA) under the following conditions: initial denaturation at 98 °C for 5 min; 30 thermal cycles at 98 °C for 30 s, 59.5 °C for 30 s and 72 °C for 45 s; and a final extension at 72 °C for 5 min. PCR amplicons were fractionated in 2% agarose gel, and target bands were extracted and purified with Vazyme VAHTS DNA Clean Beads (Vazyme, Nanjing, China). DNA concentrations were quantified using Quant-iT PicoGreen dsDNA Assay Kit (Invitrogen, CA, USA).

Amplicon libraries were constructed using TruSeq Nano DNA Library Prep Kit (Illumina, CA, USA), which included end repairing, addition of an A base at the 3'-end, adapter ligation and PCR amplification. Libraries were quantified with the Quant-iT PicoGreen dsDNA Assay Kit (Thermo Fisher Scientific, MA, USA) and prepared to a final concentration ≥ 2 nM.

### Illumina sequencing data analysis and phylogenetic reconstructions

Libraries were denatured with 0.1 N NaOH and subjected to paired-end sequencing with Illumina NextSeqTM 2000 (2 × 300 bp for 616*F/1132R amplicons; 100,000 reads per amplicon) and NovaSeq 6000 (2 × 250 bp for 1391F/EukBr and DM568F/RM2R amplicons; 100,000 and 140,000 reads per amplicon, respectively). Approximately 0.18-Gb sequence data per pooled sample, or a total of around 2.1 Gb data for the 36 pools, was obtained in this study.

Raw sequencing data were analyzed using the Quantitative Insights Into Microbial Ecology 2 (QIIME2, v2019.4) [24]. Primer sequences were trimmed with CUTADAPT (v2.3; settings: -e 0.2 and -O [minimum overlap, set to 9/10 of each primer's length]) [25]. Subsequent processing steps used VSEARCH (v2.13.4) [26]. Paired-end reads were merged using command FASTQ_MERGEPAIRS (settings: –MINOVLEN 38 –MAXDIFFS 9 for products produced by primers 616*F/1132R; –MINOVLEN 218 –MAXDIFFS 45 for 1391F/EukBr; –MINOVLEN 50 –MAXDIFFS 20 for DM568F/RM2R). Low-quality reads were removed using FASTQ_FILTER (settings: –maxee 6.9 for 616*F/1132R; –maxee 0.9 for 1391F/EukBr; –maxee 0.5 for DM568F/RM2R). The resulting sequences were dereplicated and denoised using DEREP_FULLLENGTH (–minuniquesize 2, -sizeout). Chimeric sequences were removed using UCHIME_DENOVO (–abskew 2). Clean reads were taxonomically mapped at the SILVA SSU and LSU databases (v138.2; https://www.arb-silva.de), which also produced confidence scores. The resulting annotations with high confidence (scores ≥ 0.9) were accepted, while those with low confidence scores (scores ≤ 0.5) were discarded. Annotations with moderate confidence (between 0.5 and 0.9) were re-analyzed by BLASTN searches against the GenBank nucleotide database, from which top hits with identity scores ≥ 97% and E-values < 1E − 5 were accepted. For quality control, annotations in the final datasets with < 10 reads were removed to reduce false-positive signals as proposed by Bokulich et al. [27].

In phylogenic analysis, merged clean reads derived from individual primer pairs were first examined by clustering with CD-HIT (v4.8.1; 97% similarity threshold) [28]. Unique sequences for each detected parasite species were aligned with selected homologs from GenBank or vEuPathDB databases using MAFFT (v7.505) [29] and visualized using ESPript 3.0. Maximum likelihood phylogenetic trees were inferred using MEGA (v11), with 1000 bootstrap iterations to assess node reliability. Nucleotide substitutions used the Kimura two-parameter model under uniform rate among sites.

### Validation and genotyping of *Cryptosporidium* species in pooled samples

Since *Cryptosporidium* was the major protozoan pathogen detected by 18S/28S amplicon-based NGS, we further validated the results by PCR detections and *gp60* gene subtyping for all positive pools and selected negative pools. PCR detections used three assays: (i) our recently developed TaqMan qPCR strategy, which targets *Cryptosporidium* cgd6_3920 locus for differentiate *Cryptosporidium parvum* and *C. hominis* from other species [30]; (ii) a previously established nested PCR protocol targeting the 18S rDNA locus [31]. Primary PCR used primers SSU-F2 (5'-TTC TAG AGC TAA TAC ATG CG-3') and SSU-R2 (5'-CCC ATT TCC TTC GAA ACA GGA-3') with initial denaturation at 94 °C for 3 min, 35 cycles of amplification at 94 °C for 45 s, 55 °C for 45 s and 72 °C for 60 s, and a final extension at 72 °C for 5 min. Secondary PCR used primers SSU-F3 (5'-GGA AGG GTT GTA TTT ATT AGA TAA AG-3') and SSU-R4 (5'-CTC ATA AGG TGC TGA AGG AGT A-3') with initial denaturing at 94 °C for 3 min, 35 cycles of amplification at 94 °C for 45 s, 58 °C for 45 s and 72 °C for 60 s, and a final extension at 72 °C for 5 min; (iii) a previously established nested PCR protocol targeting the *gp60* locus [32]. Each batch of PCR amplifications included negative and positive sample controls using DNA isolated from samples pre-determined to be *Cryptosporidium*-free by PCR or those spiked with the oocysts of a laboratory strain *C. parvum* (*gp60* subtype IIaA17G2R1), respectively. Primary PCR used primers were AL3531 (5'-ATA GTC TCC GCT GTA TTC-3') and AL3535 (5'-GGA AGG AAC GAT GTA TCT-3') with initial denaturing at 94 °C for 3 min, 35 cycles of 94 °C for 45 s, 52 °C for 45 s and 72 °C for 60 s, followed by a final extension at 72 °C for 5 min. Secondary PCR used primers were AL3532 (5'-TCC GCT GTA TTC TCA GCC-3') and AL3534 (5'-GCA GAG GAA CCA GCA TC-3') using the same thermal cycling conditions used for the primary PCR.

18S rRNA and *gp60* gene fragments amplified by nested PCR were analyzed with 1% agarose gel electrophoresis. DNA bands were isolated from the gels, purified using the Universal DNA Purification Kit (Tiangen, Beijing, China) and subjected to Sanger sequencing from both directions (Sangon Biotech, Shanghai, China) for validation of species and *gp60*-based subtyping.

### Indirect immunofluorescence assay (IFA) of cryptosporidial oocysts

Smears were prepared from pooled samples positive for *Cryptosporidium* after enrichment by sucrose gradient centrifugation, washes with PBS and suspension of pellets as described above. After air-drying and fixation with 10% formalin, the smears were detected using a rabbit anti-*Cryptosporidium* oocyst wall polyclonal antibody (1:500 dilution) made in house, followed by Alexa Fluor 594-labeled goat anti-rabbit IgG (1:2,000 dilution) as described [30]. Immunolabeled samples were examined under an Olympus BX52 epifluorescence microscope.

### Statistical analysis

Based on the positive detection results from pooled samples (pool size = 10, total pools = 36), true positivity rate (*pr*) of each parasite was estimated using a binomial model for pooled sampling, assuming perfect test sensitivity and specificity [33]:

$$pr=1-{(1-\frac{x}{k})}^{1/n}$$

where *x* is the number of positive pools, *k* is the total number of pools, and *n* is the number of individual samples per pool. To quantify the uncertainty in prevalence estimates, 95% confidence intervals (CIs) were derived using the profile likelihood method.

Univariate risk factor analysis was conducted to assess potential associations between parasite prevalence and categorical variables (age, sex, season and diarrhea) using Fisher’s exact test. Differences in read counts for various parasites between primer pairs were evaluated with the Chi-square test, while differences in positivity rates were evaluated using Fisher’s exact test. Two-tailed *P* values of < 0.05 were considered statistically significant.

## Results

### 18S/28S rDNA amplicon-based metabarcoding is capable of detecting intestinal parasites but limited by varied technical sensitivity and amplification biases

Metabarcoding based on 18S/28S rRNA gene (rDNA) amplicons amplified was performed for 36 pooled samples in human patients in Changchun, China. Each of the 36 pools contained an equal volume of fecal materials from 10 patients within the same category (total 360 patients). The 36 pools were divided into 12 groups categorized by age, gender and season; each group contained three biological replicates (denoted as subgroups a, b and c) (Fig. 1a). Three pairs of previously validated primers were used, including primers 616*F/1132R targeting the 18S V4V5 region, 1391F/EukBr targeting the 18S V9 region and DM568F/RM2R targeting the 28S D3D4 region.

The study produced a total of 6,088,710 clean reads (paired-end) that could be mapped to parasites and fungi at varied taxonomic levels (Table 1 and Table 2; see Additional file Table S3 for a complete list of detected parasitic and fungal species, as well as read counts). Unexpectedly, most reads from the three primer pairs were fungal (Ascomycota and Basidiomycota), constituting 98.35% of total reads; only 1.65% were from protists and helminths. The detected fungi may include pathogens, but as this study focuses on gut parasites, fungal data will not be discussed further.

**Table 1:** Summary of read counts combined from all 12 pooled samples for the three amplicons^1^

Target regions and primers		18S V4V5 616*F/1132R	18S V9 1391F/EukBr	28S D3D4 DM568F/RM2R	Subtotal	Percent of total (%)	Percent of subtotal (%)
**Product lengths (AF040725)**^2^		**509 bp**	**158 bp**	**292 bp**			
Parasites	*Cryptosporidium parvum*	33	5109	5555	10,697	0.18	10.63
	*Entamoeba hartmanni*	17,208	1135	16,715	35,058	0.58	34.84
	*Blastocystis hominis*	21,637	4406	27,090	53,133	0.87	52.80
	Liver fluke	24	1710	0	1734	0.03	1.72
Parasite subtotal		38,902	12,360	49,360	100,622	1.65	100
Fungi	Ascomycota	1,736,078	749,995	3,210,670	5,696,743	93.56	95.13
	Basidiomycota	88,400	42,007	160,938	291,345	4.79	4.87
Fungus subtotal		1,824,478	792,002	3,371,608	5,988,088	98.35	100.00
Grand total		1,863,380	804,362	3,420,968	6,088,710	100	

^1^The read counts reported here are the sum of all reads from all 12 subgroups. Read counts for individual subgroups and amplicons are summarized in Table S3

^2^Product lengths were calculated using *Cryptosporidium parvum* rRNA gene (GenBank accession number AF040725) as the reference sequence

**Table 2:** Sample groups and combined read counts from three primer sets for the four parasites (*Cryptosporidium parvum, Entamoeba hartmanni, Blastocystis hominis* and liver fluke)

Group	Code^1^	Specimen^2^	Combined rDNA amplicon reads^3^				Overall
			*C. parvum*	*E. hartmanni*	*B. hominis*	Liver fluke	
1	YMWD +	1a	650				650
		1b	11				11
		1c					
2	YMCD +	2a					
		2b					
		2c					
3	YFWD +	3a					
		3b					
		3c					
4	YFCD +	4a					
		4b			38		38
		4c					
5	MMWD −	5a					
		5b			379		379
		5c					
6	MMCD −	6a					
		6b					
		6c	13			120	133
7	MFWD −	7a	7668				7668
		7b			3007		3007
		7c					
8	MFCD −	8a					
		8b	2283		23,175		25,458
		8c					
9	OMWD −	9a	33	35,058	26,534	610	62,235
		9b					
		9c				909	909
10	OMCD −	10a					
		10b					
		10c				95	95
11	OFWD −	11a	39				39
		11b					
		11c					
12	OFCD −	12a					
		12b					
		12c					
Total reads			10,697	35,058	53,133	1734	100,622

^1^*Y* young age (0–17 years), *M* middle aged (18–60), *O* elderly (> 60 years), *M* male, *F* female, *W* warm season (April to September), *C* cold season (November to March), *D +* with diarrhea, *D–* without diarrhea. ^2^ Specimens are pooled samples from 10 patients. ^3^ Amplicon reads presented here are combined from all three primer pairs. Reads from individual primer pairs are presented in Additional file Table S3

For the detected parasites, protists could be identified into species (i.e. *Cryptosporidium parvum*, *Entamoeba hartmanni* and *Blastocystis hominis*), while helminths could only be identified at the family level (Opisthorchiidae or liver fluke) (Table 1 and Table 2). The three primer pairs produced varied technical sensitivity of detection toward the four parasite species. As discussed in more detail below, some pools (subgroups) are positive by all three primer pairs, while others are only positive by one or two primer pairs (Fig. 1a, Table S4). Additionally, while the number of amplicon read counts are indicative of abundance and commonly used in quantitative analysis, the numbers of reads for various parasite species are not proportional between those produced by the three pairs of primers (*P* < 0.001 by Chi-square test) (Fig. 1b, Table 1 and Table S3), indicating strong amplification biases between primers.

The high ratios of fungal reads, together with the varied technical sensitivity between the primers in detecting parasite species, suggest that these universal primers are not optimal for metabarcoding of parasitic species. There is a need to explore new primers that are better for metabarcoding of intestinal parasites. The apparent amplification biases between these primers also indicates that metabarcoding reads derived from these primers are not suitable for true quantitative comparison of abundances between detected taxonomic groups.

### The zoonotic *Cryptosporidium parvum* is the most prevalent pathogenic protist, followed by nonpathogenic *Blastocystis hominis* and *Entamoeba hartmanni*

*Cryptosporidium* is the most prevalent parasite detected in this study. The 28S primers DM568F/RM2R produced amplicons in six (16.67%) of the 36 pools, while 18S primers 616*F/1132R and 1391F/EukBr were each produced amplicons in two (5.56%) of the pools (Fig. 1a and Table 2). The three primer pairs produced varied technical sensitivity of detection: only one pool (mid-aged female) is positive by all three primer pairs, while one and four pools are positive by two or one of the three primer pairs (Fig. 1a and Table 2). Because of the discrepancy between primers, we consider a pool as positive when amplicons are produced by at least one of the three primer pairs, by which seven (19.44%) of the 36 pools could be judged as *Cryptosporidium*-positive. The estimated true positivity rate of *Cryptosporidium* is 2.14% (95% CI 0.92%–4.1%) using a binomial model for pooled sampling (Fig. 1c).

Of the three types of amplicons, reads produced by primers 616*F/1132R (18S V4V5) and DM568F/RM2R (28S D3D4) could be mapped to *C. parvum* at the species level, while those produced by 1391F/EukBr could only be mapped to the genus level (Table S3). Because the two positive pools by 1391F/EukBr are also positive by DM568F/RM2R, together with subsequent molecular validation and subtyping analysis (see below), we conclude that *C. parvum* is the species in the seven *Cryptosporidium*-positive pools.

**Fig. 2:**
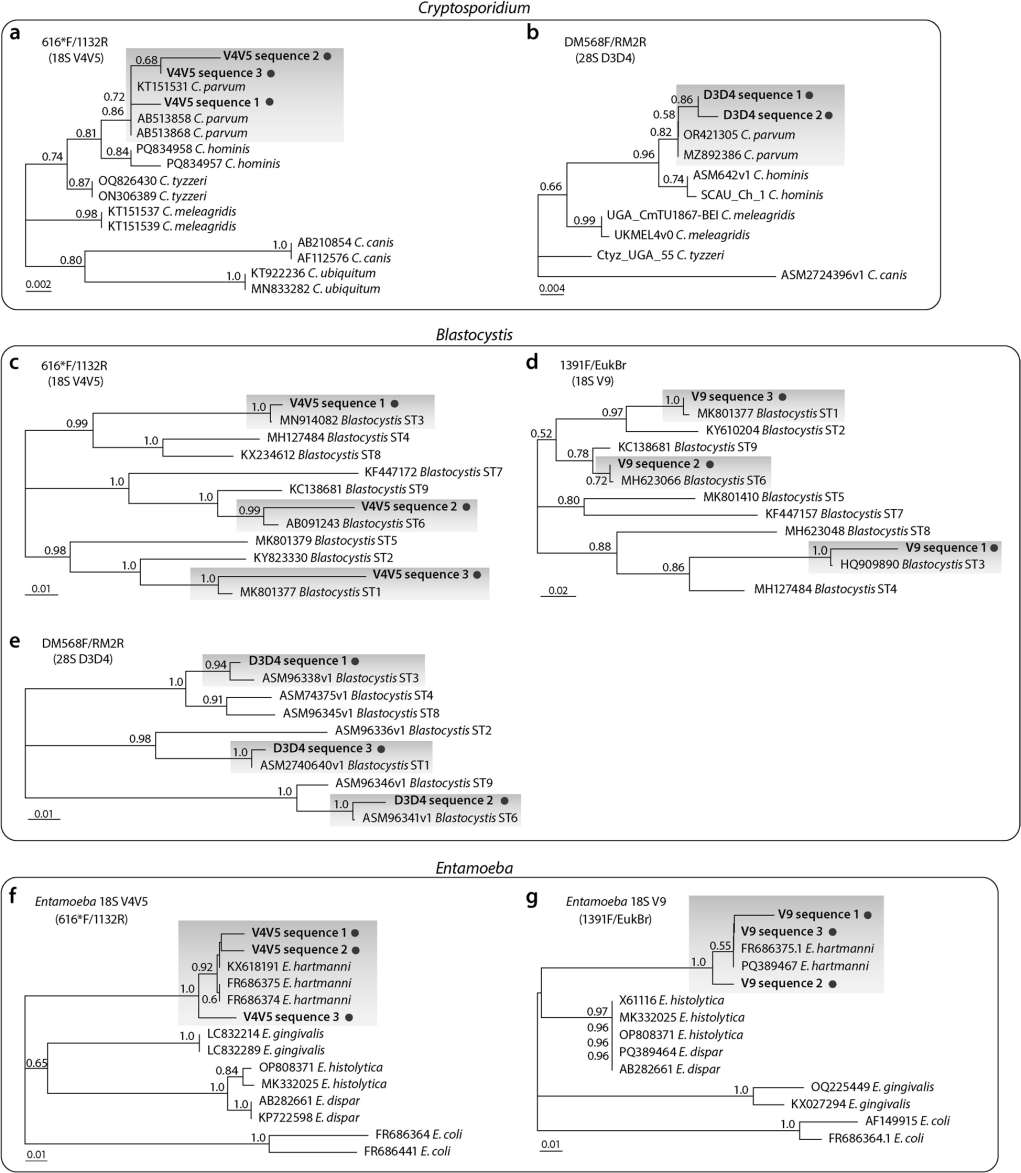
Phylogenetic trees inferred from unique 18S and 28S rRNA gene fragments for the three protists derived from metabarcoding sequences, together with closely related sequences fetched from GenBank. (**a–b**) Trees derived from *Cryptosporidium* sequences in the 18S V4V5 (**a**) and 28S D3D4 (**b**) regions. (**c-e**) Trees derived from *Blastocystis* sequences in the 18S V4V5 (c), 18S V9 (d) and 28S D3D4 (**e**) regions. (**f-g**) Trees derived from *Entamoeba* sequences in the 18S V4V5 (**f**) and 18S V9 (**g**) regions

The reads amplified from the three pairs of primers (616*F/1132R, 1391F/EukBr and DM568F/RM2R) could be assembled into three, three and two unique/consensus sequences, respectively (see additional file Fig. S1 for multiple sequence alignments). Among the three unique sequences, those derived from the primers 616*F/1132R (18S V4 region) and DM568F/RM2R (28S D3 region) could be mapped to *C. parvum* species, while that derived from primers 1391F/EukBr (18S V9 region) could only be mapped to *Cryptosporidium* at the genus level. Phylogenetic analysis shows that the two unique sequences at the 18S V4 and 28S D3 regions cluster with *C. parvum*, separating from *C. hominis* and other sequences, further confirming their species identity (Fig. 2a–b).

The non-pathogenic protist, *Blastocystis hominis*, is the second prevalent parasite, present in five (13.89%) of the 36 subgroups (Fig. 1a). The estimated true positivity rate of *B. hominis* is 1.48% (95% CI = 0.53%–3.17%) (Fig. 1c). *Blastocystis* subtype 1 (ST1) is the most prevalent, detected in three pools, while ST3 and ST6 are less prevalent, each present in one pool (Fig. 1; Fig. S2 and Table S3). In the five positive pools, four produced amplicons by all three primer pairs, while one produced amplicons by two primer pairs. Reads from all three types of amplicons could be mapped to *Blastocystis* at the subtype level (Fig. 2c-e; Fig. S2 and Table S3).

The third protist, *Entamoeba hartmanni* (a nonpathogenic amoeba), was detected in only one of the 36 pools (2.78%), especially in subgroup 9a, which consisted of older male samples. All three primer pairs identified this organism (Fig. 1; Fig. 2f–g; Table S3). The estimated true positivity rate of *E. hartmanni* is 0.28% (95% CI 0.02%–1.23%) (Fig. 1c), suggesting a relatively low level of prevalence. The two 18S amplicon reads, which were produced by primer pairs 616*F/1132R and 1391F/EukBr, could be mapped to species, while the 28S amplicon could be mapped to the genus level. Pathogenic *Entamoeba* species were not detected in this study, however. Although only one subgroup was positive of *E. hartmanni*, the read counts were high, representing 34.84% of the total parasite reads. This observations suggests that the three primers are reasonably good for amplifying amoeba sequences and/or the load(s) of *E. hartmanni* in the patient(s) is high.

### The liver fluke is prevalent in adult patients

Helminths are detected in four (11.11%) of the 36 subgroups (Fig. 1), while the amplicons could only be mapped to Opisthorchiidae (liver fluke) at the family level. The estimated true positivity rate is 1.17% (95% CI 0.36%–2.70%) (Fig. 1c). All four positive pools were detected by the 18S primers 1391F/EukBr, one of which was also positive by the 18S primers 616*F/1132R. The 28S primers did not produce helminth amplicons in any pools. Opisthorchiidae contains many flukes, in which *Clonorchis sinensis*, *Opisthorchis viverrini* and *Opisthorchis felineus* are the most medically important species. Although the species could not be determined from the metabarcoding amplicon sequences, it is likely *C. sinensis* (Chinese liver fluke), which is the only species reported in Jilin Province based on historically and recent epidemiology surveys in the region [34, 35].

Notably, 12 (33.3%) of the 36 pools are positive for one or more of the four parasites (Fig. 1). Only three (8.33%) pools are positive for multiple parasites, including one positive in four parasites in an older aged male pool (subgroups 9a) and two positive in two parasites (i.e. *C. parvum* and *B. hominis* in a middle aged female pool [subgroup 8b] and *C. parvum* and liver fluke in a middle aged male pool [subgroup 6c]). Because of using pooled samples, we are unable to conclude whether the detection of multiple parasites represents true coinfections in certain patients. Given that < 10% of the pools are positive for multiple parasites, the coinfections are low if present.

### No significant risk factors were associated with the metabarcoding-based parasite detection in pooled samples

Although the study design aimed to balance the statistical power by including three pools (biological replicates) for each of 12 patient groups defined by age, gender and season, Fisher’s exact test did not identify any factors significantly associated with the prevalences (positive detection of DNA) of individual parasites or with the overall prevalence of any parasites in the pooled samples (Table 3). While no associations reached statistical significance, it is noteworthy that the young age subgroups tested positive only for *C. parvum* (*n* = 2) and *B. hominis* (*n* = 1). In contrast, the mid-age and older subgroups had more positives for *B. hominis* and liver fluke, including one mid-aged pool (subgroup 6c) positive for both *C. parvum* and liver fluke and an older age pool for all four parasites (subgroup 9a).

**Table 3:** Risk factor analysis by Fisher’s exact test

Parasite	Variable	Category	Positive N	Negative N	Positive rate (%)	*P*-value
*Cryptosporidium parvum*	Age	Young	2	10	16.7	1
		Mid-aged	3	9	25.0	
		Older	2	10	16.7	
	Sex	Male	4	14	22.2	1
		Female	3	15	16.7	
	Season	Warm	5	13	27.8	0.4
		Cold	2	16	11.1	
	Diarrhea	Yes	2	10	16.7	1
		No	5	19	20.8	
*Blastocystis hominis*	Age	Young	1	11	8.3	1
		Mid-aged	3	9	25.0	
		Older	1	11	8.3	
	Sex	Male	2	16	11.1	1
		Female	3	15	16.7	
	Season	Warm	3	15	16.7	1
		Cold	2	16	11.1	
	Diarrhea	Yes	1	11	8.3	0.65
		No	4	20	16.7	
Liver fluke	Age	Young	0	12	0	0.29
		Mid-aged	1	11	8.3	
		Older	3	9	25.0	
	Sex	Male	4	14	22.2	0.10
		Female	0	18	0	
	Season	Warm	2	16	11.1	1
		Cold	2	16	11.1	
	Diarrhea	Yes	0	12	0	0.27
		No	4	20	16.7	
Any parasites	Age	Young	3	9	25.0	0.90
		Mid-aged	5	7	41.7	
		Older	4	8	33.3	
	Sex	Male	7	11	38.9	0.72
		Female	5	13	27.8	
	Season	Warm	8	10	44.4	0.29
		Cold	4	14	22.2	
	Diarrhea	Yes	3	9	25.0	0.71
		No	9	15	37.5	

The diarrhea symptom is not expected to be significantly associated with any of the parasites, particularly *C. parvum*, although that the samples are not random for the diarrhea factors (i.e. young age groups are all diarrhea patients, whereas the other two age groups are all non-diarrhea patients; see the explanation in Materials and Methods). These observations imply that, while *C. parvum* is emerging as a significant intestinal protozoan parasite in the northeast China, it was not the major cause of diarrhea in children who visited the hospital.

### IIdA19G1 is the major subtype of *C. parvum* in patients of all three age groups

**Fig. 3:**
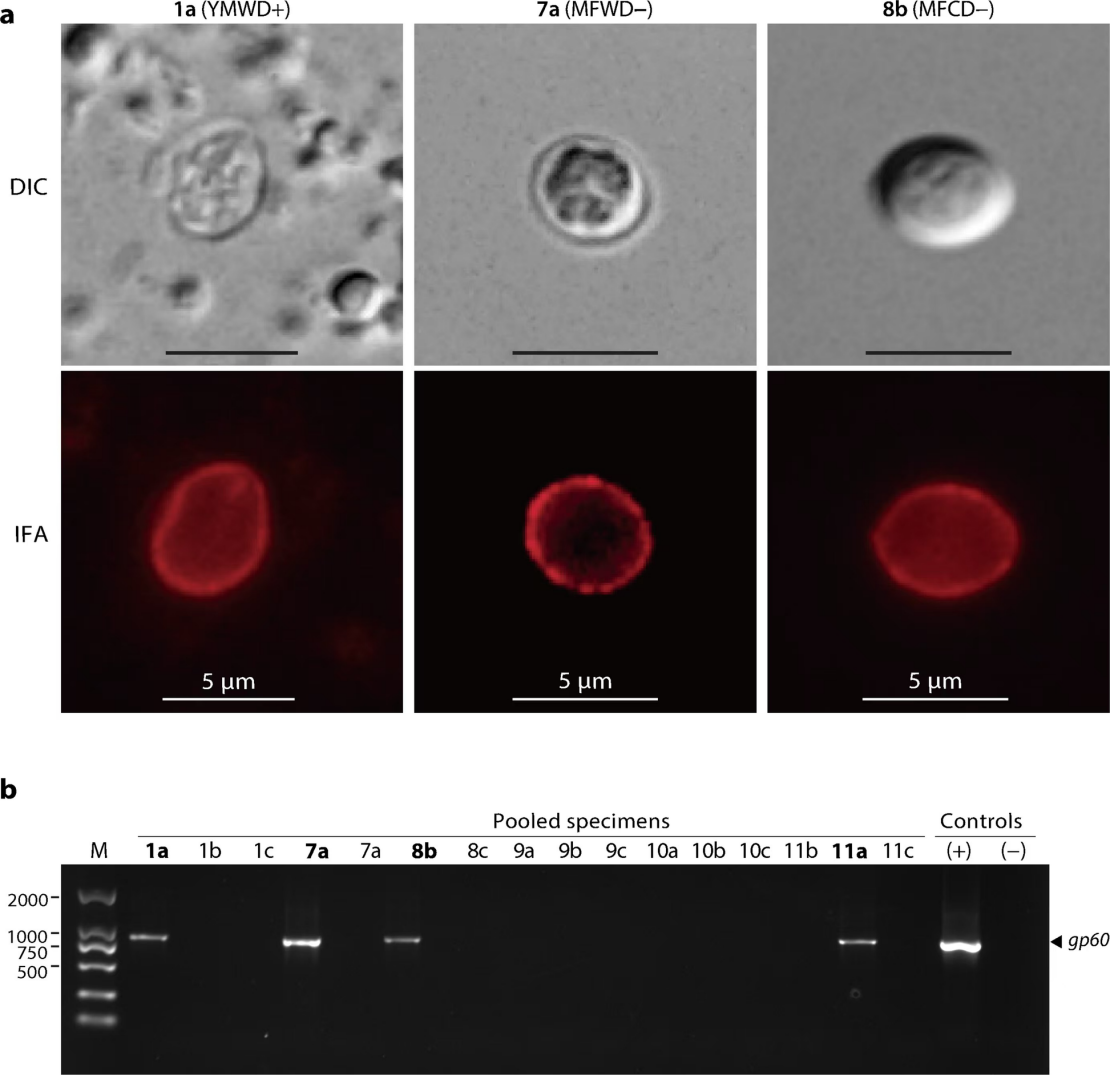
Microscopic detection of *Cryptosporidium parvum* oocysts and PCR detection of *C. parvum gp60* gene. **a** Micrographs of oocysts observed from specified pools (sample subgroups). DIC, differential interference contrast microscopy; IFA, immunofluorescence assay using a polyclonal antibody against a *Cryptosporidium* oocyst wall protein. **b** Agarose gel electrophoresis showing the positive and negative detection of *C. parvum gp60* gene fragments from selected pools. PCR bands from positive pools were sequenced for genome subtyping as described in the manuscript

To further validate the cryptosporidial infection and gain more details about the the molecular features, we performed IFA in pooled fecal pools enriched by sucrose gradient centrifugation using a polyclonal antibody against an oocyst wall protein, confirming the presence of oocysts in three pools (Fig. 3a; Table S3). We also detected *C. parvum* from four pools by qPCR targeting the cgd6_3920 gene and by two nested PCR targeting the 18S rRNA and *gp60* genes. The species are further confirmed as *C. parvum* by sequencing of the PCR products. The sequence of *gp60* amplicons also confirms that IIdA19G1 is the subtype in the four *C. parvum*-positive pools (Fig. 3b; Table 4).

**Table 4:** Molecular validation and subtyping of *Cryptosporidium* species in selected positive specimens and detection of oocysts by immunofluorescent assay

Specimen code	qPCR on cgd6_3920	Nested PCR on 18S rDNA	Nested PCR and subtyping on *gp60*	Oocysts observed by IFA
1a (YMWD +)	*C. parvum*	*C. parvum*	IIdA19G1	Yes
7a (MFWD −)	*C. parvum*	*C. parvum*	IIdA19G1	Yes
8b (MFCD −)	*C. parvum*	*C. parvum*	IIdA19G1	Yes
11a (OFWD −)	*C. parvum*	*C. parvum*	IIdA19G1	Not observed

## Discussion

This study aimed to provide a snapshot survey of intestinal parasites in humans in Changchun city in the northeast of China, for which current knowledge is Limited. We intended to use high-throughput molecular approaches with the advantage for simultaneous detection of multiple species. We initially performed a small-scale study to compare the effectiveness of detection between metagenomic and 18S/28S amplicon-based metabarcoding approaches and observed that, under similar sequencing depth, metabarcoding was more sensitive than metagenomics in detecting parasites in fecal samples (data not shown). The metagenomic method detected fewer parasite species with few reads, as most metagenomic reads were derived from bacteria even after rough enrichment by sucrose floatation.

Several primers targeting various regions in 18S and 28S rDNA were reported in amplicon-based metabarcoding [36–38]. Based on a previous evaluation and recommendation [21], we selected three primer pairs, in which two target 18S V4V5 (primers 616*F/1132R) and V9 (1391F/EuBr) regions and one targets 28S D3 region (DM568F/RM2R). The use of multiple pairs of primers was intended to increase the effectiveness of detection, balance the amplification bias between primers and produce more sequence information to improve taxonomic resolution. To further increase the technical sensitivity of detection, we performed a sucrose-based floatation to enrich oocysts, cysts and eggs, followed by freeze-and-thaw cycles and high-speed beating with steel beads to ensure the release of genetic materials.

The approaches allow us to detect various parasites, but the fungal read counts are overly high, representing > 98% of the reads in parasite-positive pools. The amplification of fungal reads is expected when pan-eukaryotic primers are used, as mycobiomes are commonly present in human gastrointestinal tracts [39–43]. However, it is unclear whether these primers are more biased towards fungal sequences over parasites. Nonetheless, our data suggest that the currently used primers are not optimal for detecting parasites. Additionally, the ratios of read counts between parasite species are also highly disproportional among the three types of amplicons (*P* < 0.0001 by Chi-square test) (Fig. 1b). This implies strong amplification biases between the primers, and the read counts produced by any single primer pair are not reliable for quantitative assessment of abundances between parasites. Overall, there is a need to explore new primers more optimal or favorable for metabarcoding of intestinal parasites.

This study detected four parasites in patient fecal samples, in which the zoonotic *C. parvum* (19.4% in pooled samples) is the most prevalent, followed by *B. hominis* (13.9%), liver fluke (11.1%) and *E. hartmanni* (2.8%). The zoonotic *C. parvum* has been recognized as a globally important parasite, responsible for frequent waterborne outbreaks [6, 44]. It is one of the major agents causing opportunistic infections in immunocompromised individuals [45]. It is also one of the major agents responsible for severe to deadly neonatal calf diarrhea. *Cryptosporidium* in China are more frequently reported in animals than in humans, including those from northeast China. The estimated prevalence (2.14%) based on the positive detection of pooled samples in this study was comparable to those reported by other investigators (e.g. 0.26–3.9% in humans) [46–50].

This study identified IIdA19G1 as the only subtype of *C. parvum* in the human samples, which has been previously reported in humans and cattle in China. It was first reported in 2019 in Zhengzhou (central China; four hospitalized children) and later in 2022 in Heilongjiang (another city in northeast China; one villager) and in 2024 in Wenzhou (southeast China; 14 children attending a children’s hospital) [50–52]. In two large-scale comparative *C. parvum* genomics studies reported in 2022 and 2024 [53, 54], IIdA19G1 was identified as the most common cattle subtype in China; however, the studies did not include any human isolates in China. Outside of China, IIdA19G1 subtype was only reported in 2016 in one out of the 110 symptomatic *Cryptosporidium*-positive patients attending two major public hospitals in Madrid, Spain [55]. This study confirms the distribution of IIdA19G1 subtype in both children and adults in Changchun (northeast China), suggesting that this subtype of *C. parvum* is emerging as one of the major intestinal protozoan pathogens in humans in China. IIdA19G1 subtype has also been frequently reported in various animals in China, including livestock and wild animals (e.g. [44, 56–58]). A more recent study also detected IIdA19G1 as one of the subtypes in river water in Changchun [30]. These show the establishment of a zoonotic transmission cycle for the *C. parvum* IIdA19G1 subtype in China.

*Blastocystis* is more frequently reported in humans, in which subtype ST3 is predominant, but ST1 and ST6 are also common [59]. In this study, ST1 was the most prevalent (*n* = 3), followed by ST3 and ST6 (each *n* = 1), implying that the population structure of *Blastocystis* in the northeast of China might differ from those in other regions. Only one pool was positive for *Entamoeba hartmanni*, which is a non-pathogenic amoeba parasite. Notably, some other common intestinal protists were not detected in this study, including non-pathogenic *Entamoeba dispar* and the pathogenic *E. histolytica*, as well as *Giardia*.

Notably, the fish-borne Liver fluke was detected in four out of the 36 pooled samples. Human liver fluke (*C. sinensis*) is relatively prevalent in this region, attributed to the dietary practice of consuming raw freshwater fish among certain population groups. In a study reported in 2020, a cross-sectional survey conducted in villages along the Lalin River revealed a high prevalence rate of 29.3% among residents, with principal risk factors linked to the consumption of raw freshwater fish [34]. Some other common intestinal helminths were not detected, such as the soil-transmitted helminths like *Ascaris* and *Trichuris*. Although there is a lack of recent data in the region, a 2017 survey of inhabitants in Yanbian Prefecture, Jilin Province, detected low positivity rates of *Ascaris lumbricoides* (0.8%), hookworm (0.06%) and *Trichuris trichiura* (0.02%) [60]. This implies that, in modern-day large cities in northeast China, food-transmitted parasites remain a major public health concern. It is also possible that the primers were not optimal for metabarcoding of intestinal helminths, which is partially supported by the fact that only one of the three pairs of primers produced amplicons from the four liver fluke-positive pools, and only one of the other two pairs of primers produced amplicons from one of the four positive pools. Additionally, while pooling samples is cost-effective, it can mask individual variation and dilute parasite loads, reducing detection sensitivity and specificity. To address these limitations, future experimental designs might be improved by adjusting pool sizes and sequencing depths and by optimizing primers for intestinal parasites.

## Conclusion

In summary, our 18S/28S rRNA‑gene metabarcoding survey provides the first molecular portrait of intestinal parasites circulating among hospital patients in Changchun. The data confirm the zoonotic *C.* *parvum* subtype IIdA19G1 as the dominant protozoan pathogen and capture a background of non‑pathogenic *Blastocystis* and amoeba, alongside evidence of persistent liver fluke exposure in adults. While the approach successfully detected low‑abundance taxa from pooled material, the pronounced primer‑ and template‑dependent biases we observed caution against direct quantitative interpretation of amplicon counts. Future surveillance should combine optimized, parasite‑focused primer sets with targeted qPCR and, where possible, metagenomic sequencing to validate prevalence estimates and explore transmission dynamics. Such integrated molecular surveillance will be essential for developing One‑Health control strategies that address zoonotic and food‑borne parasites in Northeast China and beyond.

## Supplementary Information

Additional file 1. Table S1. List of PubMed-indexed original research articles concerning the epidemiology of intestinal parasites in humans and/or animals in China from 2015 to 2025

Additional file 2. Table S2. Information of deidentified patient information and grouping from which fecal samples were collected

Additional file 3. Table S3. Detailed metabarcoding results and read counts of *Cryptosporidium*, *Entamoeba*, *Blastocystis* and liver fluke, as well as fungi, by groups/subgroups and individual primer pairs. Read counts are in parentheses.

Additional file 4. Table S4. Fisher’s exact test comparison of three 18S/28S primer pairs for metabarcoding detection of intestinal parasites

Additional file 5. Fig. S1. Multiple sequence alignments of *Cryptosporidium* unique sequences obtained in this study with selected reference sequences from GenBank.

Additional file 6. Fig. S2. Multiple sequence alignments of *Blastocystis* unique sequences obtained in this study with selected reference sequences from the GenBank.

Additional file 7. Fig. S3. Multiple sequence alignments of *Entamoeba* unique sequences obtained in this study with selected reference sequences from the GenBank.

## Data Availability

The raw sequencing data generated in this study have been deposited in the figshare repository under accession DOI [10.6084/m9.figshare.28503410]. The dataset includes FASTQ files for all 36 samples. Nucleotide sequences used for multiple sequence alignments, phylogenetic analysis and genotyping are deposited in the GenBank database under the following accession numbers: PV789538-PV789540 (18S) and PV789567-PV789568 (28S) for *C. parvum* rRNA gene fragments; PV789541-PV789543, PV789553-PV789555 (18S) and PV789569-PV789571 (28S) for *B. hominis* rRNA gene fragments; PV789545-PV789547, PV789556-PV789558 (18S) for *E. hartmanni* rRNA gene fragments and PV797445-PV797448 for *C. parvum gp60* gene fragments.
