# Supplementary material for: 18S/28S rDNA metabarcoding identifies Cryptosporidium parvum and Blastocystis ST1 as the predominant intestinal protozoa in hospital patients from Changchun, Northeast China
Source: Parasit Vectors. 2025 Sep 24;18:376. doi: 10.1186/s13071-025-07043-z (PMC12462306; doi:10.1186/s13071-025-07043-z)
Supplement: Supplementary file 1 — Additional file 1. Table S1. List of PubMed-indexed original research articles concerning the epidemiology of intestinal parasites in humans and/or animals in China from 2015 to 2025 [file 13071_2025_7043_MOESM1_ESM.pdf]

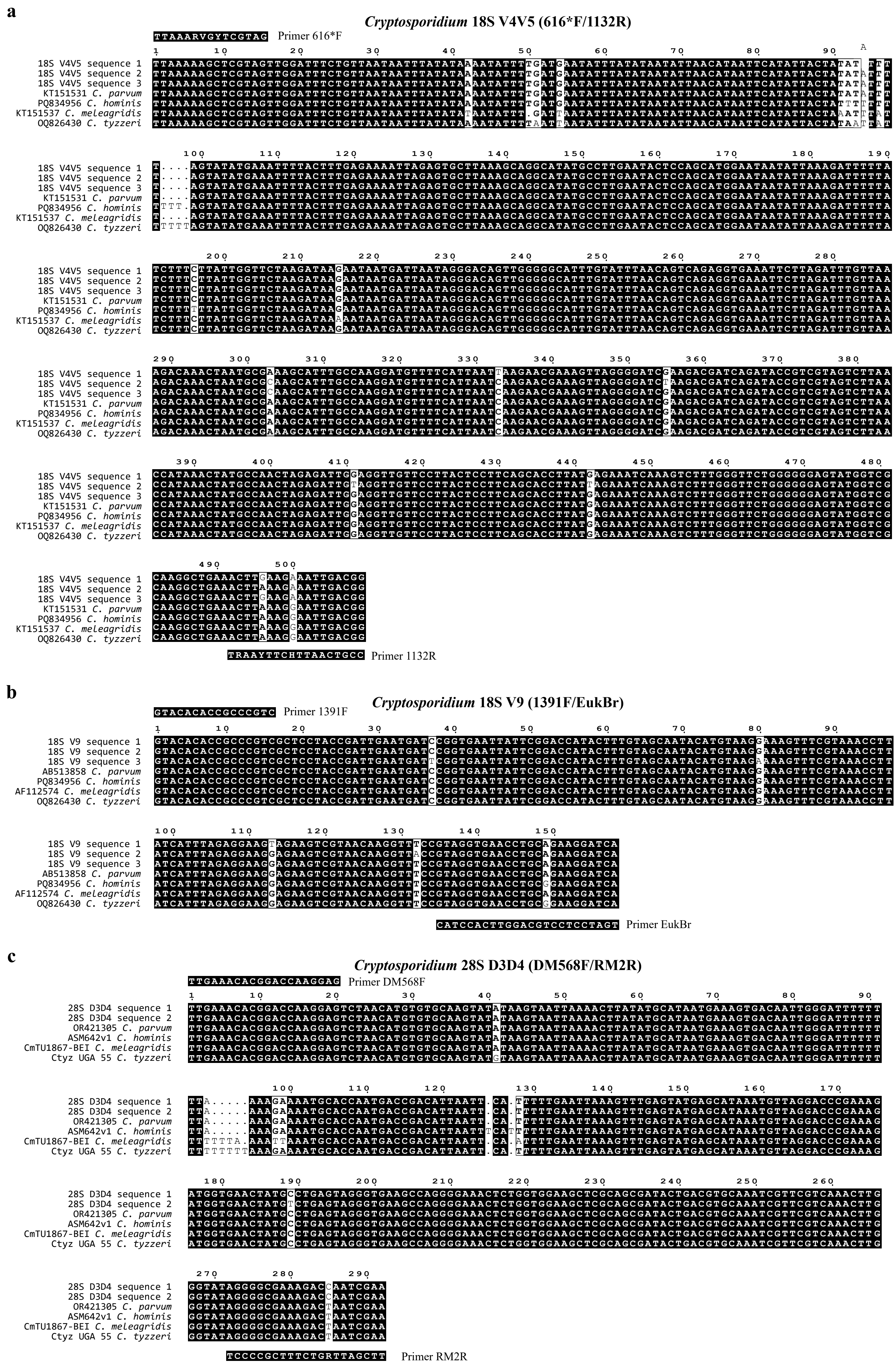
a
Cryptosporidium 18S V4V5 (616*F/1132R)
TTAAARVGYTCGTAG Primer 616*F
18S V4V5 sequence 1
18S V4V5 sequence 2
18S V4V5 sequence 3
KT151531 C. parvum
PQ834956 C. hominis
KT151537 C. meleagridis
OQ826430 C. tyzzeri
TRAAYTTCHTTAACTGCC Primer 1132R
b
Cryptosporidium 18S V9 (1391F/EukBr)
GTACACACCGCCCGTC Primer 1391F
18S V9 sequence 1
18S V9 sequence 2
18S V9 sequence 3
AB513858 C. parvum
PQ834956 C. hominis
AF112574 C. meleagridis
OQ826430 C. tyzzeri
CATCCACTTGGACGTCCTCCTAGT Primer EukBr
c
Cryptosporidium 28S D3D4 (DM568F/RM2R)
TTGAAACACGGACCAAGGAG Primer DM568F
28S D3D4 sequence 1
28S D3D4 sequence 2
OR421305 C. parvum
ASM642v1 C. hominis
CmTU1867-BEI C. meleagridis
Ctyz UGA 55 C. tyzzeri
TCCCCGCTTTCTGRTTAGCTT Primer RM2R
