# Supplementary material for: 18S/28S rDNA metabarcoding identifies Cryptosporidium parvum and Blastocystis ST1 as the predominant intestinal protozoa in hospital patients from Changchun, Northeast China
Source: Parasit Vectors. 2025 Sep 24;18:376. doi: 10.1186/s13071-025-07043-z (PMC12462306; doi:10.1186/s13071-025-07043-z)
Supplement: Supplementary file 2 — Additional file 2. Table S2. Information of deidentified patient information and grouping from which fecal samples were collected [file 13071_2025_7043_MOESM2_ESM.pdf]

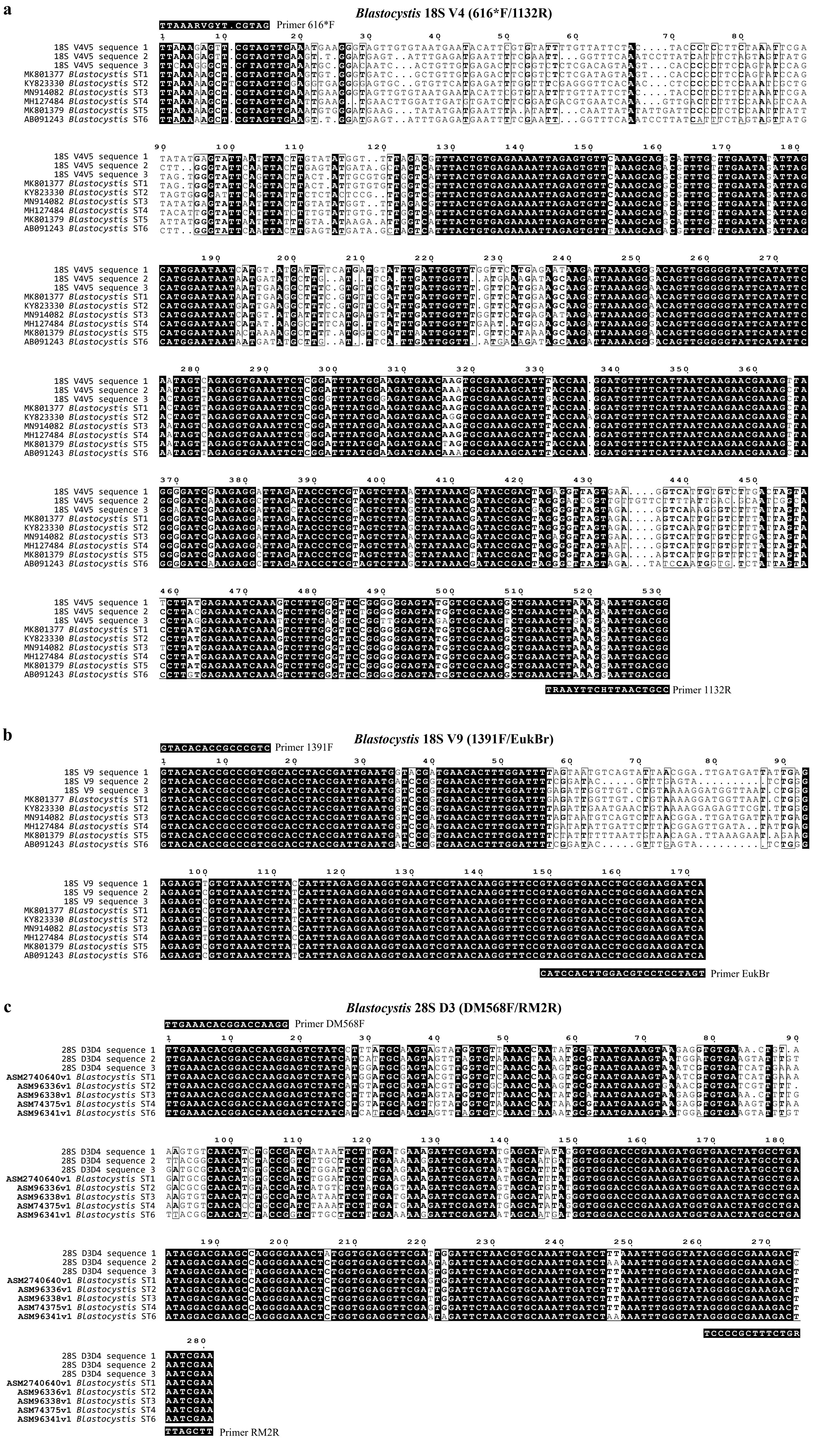
a
Blastocystis 18S V4 (616*F/1132R)
TTAAARVGYT.CGTAG Primer 616*F
TRAAYTTCHTTAACTGCC Primer 1132R
b
Blastocystis 18S V9 (1391F/EukBr)
GTACACACCGCCCGTC Primer 1391F
CATCCACTTGGACGTCCTCCTAGT Primer EukBr
c
Blastocystis 28S D3 (DM568F/RM2R)
TTGAAACACGGACCAAGG Primer DM568F
TCCCCGCTTTCTGR
TTAGCTT Primer RM2R
