# Supplementary material for: 18S/28S rDNA metabarcoding identifies Cryptosporidium parvum and Blastocystis ST1 as the predominant intestinal protozoa in hospital patients from Changchun, Northeast China
Source: Parasit Vectors. 2025 Sep 24;18:376. doi: 10.1186/s13071-025-07043-z (PMC12462306; doi:10.1186/s13071-025-07043-z)
Supplement: Supplementary file 3 — Additional file 3. Table S3. Detailed metabarcoding results and read counts of Cryptosporidium, Entamoeba, Blastocystis and liver fluke, as well as fungi, by groups/subgroups and individual primer pairs. Read counts are in parentheses. [file 13071_2025_7043_MOESM3_ESM.pdf]

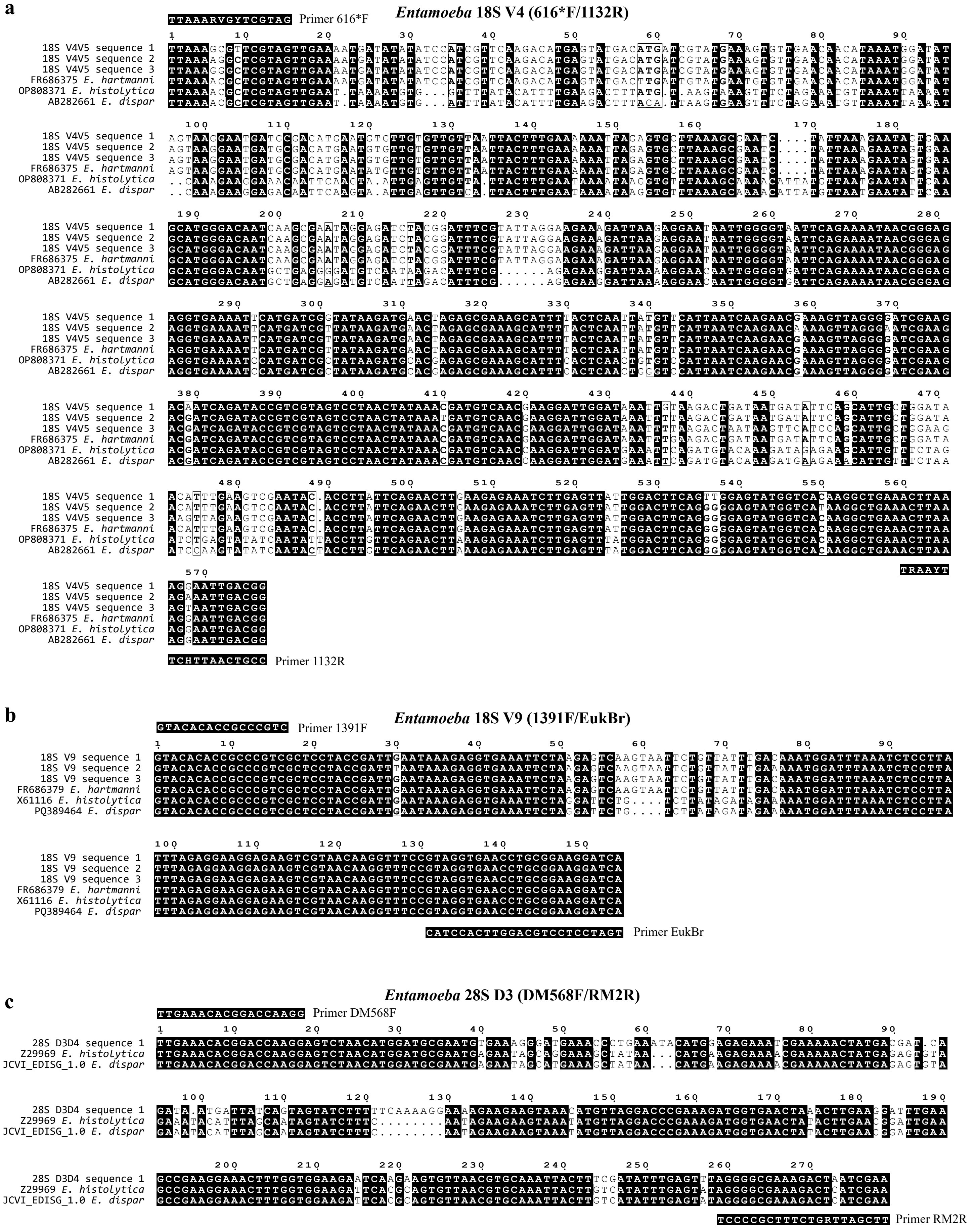
a
Entamoeba 18S V4 (616*F/1132R)
TTAAARVGYTCGTAG Primer 616*F
18S V4V5 sequence 1
18S V4V5 sequence 2
18S V4V5 sequence 3
FR686375 E. hartmanni
OP808371 E. histolytica
AB282661 E. dispar
TRAAYT
TCHTTAACTGCC Primer 1132R
b
Entamoeba 18S V9 (1391F/EukBr)
GTACACACCGCCCGTC Primer 1391F
18S V9 sequence 1
18S V9 sequence 2
18S V9 sequence 3
FR686379 E. hartmanni
X61116 E. histolytica
PQ389464 E. dispar
CATCCACTTGGACGTCCTCCTAGT Primer EukBr
c
Entamoeba 28S D3 (DM568F/RM2R)
TTGAAACACGGACCAAGG Primer DM568F
28S D3D4 sequence 1
Z29969 E. histolytica
JCVI_EDISG_1.0 E. dispar
TCCCCGCTTTCTGRTTAGCTT Primer RM2R
