# Supplementary material for: 18S/28S rDNA metabarcoding identifies Cryptosporidium parvum and Blastocystis ST1 as the predominant intestinal protozoa in hospital patients from Changchun, Northeast China
Source: Parasit Vectors. 2025 Sep 24;18:376. doi: 10.1186/s13071-025-07043-z (PMC12462306; doi:10.1186/s13071-025-07043-z)
Supplement: Supplementary file 7 — Additional file 7. Fig. S3. Multiple sequence alignments of Entamoeba unique sequences obtained in this study with selected reference sequences from the GenBank. [file 13071_2025_7043_MOESM7_ESM.pdf]

Table S4. Fisher's exact test comparison of three 18S/28S primer pairs for metabarcoding detection of intestinal parasites

Parasite	Primer pairs	Positive N	Negative N	Positive rate (%)	*P*-value
Cryptosporidium parvum	616*F/1132R	2	34	0.06	0.211
	1391F/EukBr	2	34	0.06	
	DM568F/RM2R	6	30	0.17	
Entamoeba hartmanni	616*F/1132R	1	35	0.03	1.000
	1391F/EukBr	1	35	0.03	
	DM568F/RM2R	1	35	0.03	
Blastocystis hominis	616*F/1132R	5	31	0.14	1.000
	1391F/EukBr	5	31	0.14	
	DM568F/RM2R	4	32	0.11	
Liver fluke	616*F/1132R	1	35	0.03	0.124
	1391F/EukBr	4	32	0.11	
	DM568F/RM2R	0	36	0.00	
